# Selective Strength Training Changes the Morphology and Ankle Strength of the Peroneus Longus and the Peroneus Brevis

**DOI:** 10.5114/jhk/176131

**Published:** 2024-02-17

**Authors:** Satoshi Arima, Noriaki Maeda, Sakura Oda, Yuki Tamura, Makoto Komiya, Tsubasa Tashiro, Yukio Urabe

**Affiliations:** 1 Department of Sports Rehabilitation, Graduate School of Biomedical and Health Sciences, Hiroshima University, Hiroshima, Japan.

**Keywords:** muscle strength training, peroneus muscles, foot, ankle, ultrasound imaging system

## Abstract

This study aimed to investigate the 8-week selective training effect of the peroneus longus (PL) and the peroneus brevis (PB) on muscle morphology, echogenicity, and ankle strength and to examine post-intervention detraining effects. Twenty healthy participants without orthopedic disease in the lower extremities were assigned to either the PL intervention group (training consisted of pushing the Thera-Band® out from the ball of the foot to emphasize ankle eversion) or the PB intervention group (training consisted of pulling the Thera-Band® from the base of the fifth metatarsal to enhance ankle valgus and external rotation). Each intervention was performed three times per week for 8 weeks. The cross-sectional area (CSA), thickness, echogenicity, and ankle strength of the PL and the PB were measured before week 1 and after each training session. Detraining effects were evaluated after the 8-week intervention. The results revealed a significant interaction between within-group (week) and between-group (type of intervention) variables on CSA and ankle strength of both the PL and the PB. Over the 8-week training period, the CSA and ankle strength of the PL significantly increased in the PL intervention group, as did the CSA and ankle strength of the PB in the PB intervention group (p < 0.05). The residual effect of muscle hypertrophy was observed during the detraining period. In conclusion, 8-week selective PL and PB training interventions can increase the CSA and ankle strength of these muscles over time. Long-term selective intervention is required to improve peroneus muscle morphology and function, with separate assessments of the CSA and ankle strength of the PL and the PB.

## Introduction

The peroneus muscles are major muscles acting on ankle eversion (Seo and Lee, 2022) that contribute to lateral ankle stability (Bamber et al., 2021). The main peroneal muscles are the peroneus longus (PL) and the peroneus brevis (PB), each with specific roles. For example, the PL pulls the base of the first metatarsal to stabilize the medial longitudinal arch and improve foot arch stiffness (Kirby, 2017). Conversely, the PB is inserted into the fifth metatarsal and contributes to the stability of the fifth ray (Jarrett et al., 2020). Thus, the PL and the PB contribute to ankle stability and foot arch stiffness by exerting different roles. Furthermore, low PL activity has been reported to be related to flat foot, a common foot condition (Murley et al., 2009; Saleh et al., 2021). Moreover, decreased function of the PB results in decreased stability of the fifth ray, leading to fractures of the fifth metatarsal, hypermobility, and ankle sprain, which occur frequently in sports activities (Jarrett et al., 2020; Kopytko et al., 2023). These reports indicate that dysfunction of the PL and the PB can cause dissimilar problems in the foot and the ankle. Therefore, the roles of the PL and the PB should be considered independently in treating foot and ankle issues, as well as in injury prevention and improving muscle function in healthy individuals.

Strength training of the peroneal muscles increases ankle strength and stability and effectively improves muscle function (Wang et al., 2021). The one-legged bridge exercise with ankle plantar flexion has been reported to increase PL activity because the weight concentrated on the ball of the foot is supported by the first ray to which the PL is attached (Schunk, 1982). Additionally, when the PL and the PB apply the same load to their respective muscles in the early heel rise phase, in which the PL and the PB are most active during the gait, the subtalar joint shows more valgus and external rotation when the PB is loaded than when the PL is loaded (Otis et al., 2004). These findings suggest that foot and ankle exercises emphasizing the ball of the foot loading for the PL and ankle exercises emphasizing valgus and external rotation for the PB may increase selective muscle activity.

Moreover, resistance training is also an effective way of increasing muscle mass, with resistance training of at least 6 weeks to achieve muscle hypertrophy (Damas et al., 2018). Furthermore, training has a residual effect on the muscle cross-sectional area (CSA) and strength during the detraining period after resistance training (Fatouros et al., 2005; Mujika and Padilla, 2001). Therefore, the residual effect of muscle hypertrophy and muscle strength improvement during the detraining period should be considered and measured to evaluate training effectiveness.

Recently, ultrasound imaging systems have been used to confirm the effects of long-term strength training on muscle hypertrophy (Ribeiro-Alvares et al., 2018) and to measure the PL and PB CSA separately (Arima et al., 2022). The two measurement locations were 25% proximal to the line joining the fibula head and lateral malleolus, where most of the CSA of the PL is located, and 75% distal to this line for the CSA of the PB (Arima et al., 2022). This was based on previous studies examining the anatomical location of the PL and PB muscles (Lee et al., 2011). Muscle echogenicity indicates the presence of adipose or fibrous tissue infiltration within the muscle; when such tissue is present, the CSA of the muscle appears white and bright on ultrasound images (Ong et al., 2017). Thus, if the muscle is hypertrophied and the percentage of muscle fibers in the CSA increases, it will appear darker on the ultrasound image, thereby reflecting muscle hypertrophy. Consequently, measuring muscle morphology and echogenicity over time at the proximal 25% and distal 75% locations may be helpful to assess muscle hypertrophy during long-term strength training for the PL and the PB. However, there have been no reports of long-term strength training interventions separately for the PL and the PB.

Here, we aimed to determine whether 8 weeks of selective training for the PL and the PB would result in differential alterations in muscle morphology, echogenicity, and ankle strength of both muscles over time and to examine the long-term training effects by assessing the residual effects of the detraining period. We hypothesized that implementing an intervention with the ball of the foot load awareness would increase the CSA and thickness of the PL and the ball of the foot-aware ankle strength over the course of the training weeks. Regarding the valgus and ankle external rotation-aware intervention, we hypothesized that the CSA and thickness of the PB and the ankle valgus and external rotations-aware ankle strength would improve over the course of 8-week training. Finally, we hypothesized that muscle echogenicity value would decrease with each time course for both interventions in a manner dependent on the ball of the foot loading and the ankle’s valgus and external rotation.

## Methods

### Participants

This study followed an intervention design and included 20 healthy adults (10 males and 10 females) whose general information is summarized in Table 1. The inclusion criteria encompassed healthy adults with no history of orthopedic disorder in the lower extremities. The following exclusion criteria were set: (1) a history of lateral ankle sprain and other orthopedic conditions or lower-extremity orthopedic surgery; (2) patients who experienced an acute musculoskeletal injury such as a fracture or sprain of the lower extremity within the past three months; (3) a Cumberland Ankle Instability Tool score of 27 or less (Forsyth et al., 2022); and (4) competitive athletes and those engaged in systematic resistance training. The research protocol complied with principles embodied in the Declaration of Helsinki and was approved by the appropriate ethical review board. The purpose and content of this study were fully explained to the participants in accordance with the Declaration of Helsinki, followed by obtaining written informed consent. The study was also registered as a clinical trial in the University Hospital Medical Information Network-Clinical Trials Registry and underwent ethical review and approval.

**Table 1:** General information of participants (n = 20, male: n = 10, female: n = 10).

	PL group (n = 10)	PB group (n = 10)	*p*-value
Age (years old)	22.7±1.6	22.5±1.4	0.77
Height (cm)	162.4±7.3	164.4±7.1	0.54
Body weight (kg)	55.7±6.1	59.5±10.3	0.33
Body Mass Index (kg/m^2^)	21.0±0.9	21.9 ± 2.7	0.36
The number of ankle sprains (times)	0.0±0.0	0.0±0.0	-
CAIT score	28.9±1.9	29.3±1.0	0.56

Mean ± SD, CAIT: Cumberland ankle instability tool, PL: Peroneus longus, PB: Peroneus brevis

### Experimental Procedures

**Figure 1:**
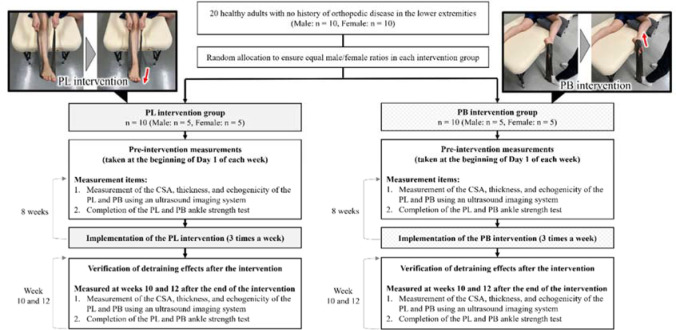
Flowchart of the methodology of the study. PL: peroneus longus, PB: peroneus brevis, CSA: cross-sectional area

A flowchart of the methodology employed in this study is shown in Figure 1. Participants were randomly divided into two groups, the PL and PB intervention groups, with equal proportions of males and females. In the PL intervention group, the Thera-Band^®^ (strength, level +3 [black]; Hygienic Corporation, Akron, OH, USA) was applied to the ball of the foot in the long sitting position and pushed out from the attachment point at maximum ankle plantar flexion. During the PL intervention, the Thera-Band^®^ with no tension was set in the plantar foot in the mid-ankle position, and both its ends were half the length from the fibular head to the plantar foot. The intervention was performed with the ends of the Thera-Band pulled up to the knee joint space.

Furthermore, in the PB group, the intervention was performed in the lateral position with the Thera-Band^®^ pulled out from the base of the fifth metatarsal, enhancing the ankle’s valgus and external rotation at maximum ankle plantar flexion. As an initial setting, the Thera-Band^®^ was adjusted to 50 cm, the same height as that of the bed used for the intervention, with no tension applied to it and both ends joined together. Each participant completed the PL or PB intervention three times a week for a total of 8 weeks. To standardize the load applied during training, the length of the Thera-Band^®^ was determined for each participant and intervention. The lateral position was set at 90° of the hip and knee flexion for the right lower extremity and 0° of the hip and knee flexion/extension for the left lower extremity. The two interventions were conducted in two sets of 100 repetitions at a speed of 1 repetition/2 s. All exercises were performed with the right leg only. Prior to the interventions at week 1 (baseline) and on the first day of each subsequent week of the intervention, the CSA, thickness, and echogenicity at the proximal 25% and distal 75% locations were measured using an ultrasound imaging system. The ankle strength tests of the PL and the PB were conducted using a handheld dynamometer. To evaluate the post-intervention detraining effects, the same measurements were conducted once at weeks 10 and 12 after the end of the intervention period.

### Measurement of PL and PB Morphology and Echogenicity Using the Ultrasound Imaging System

**Figure 2:**
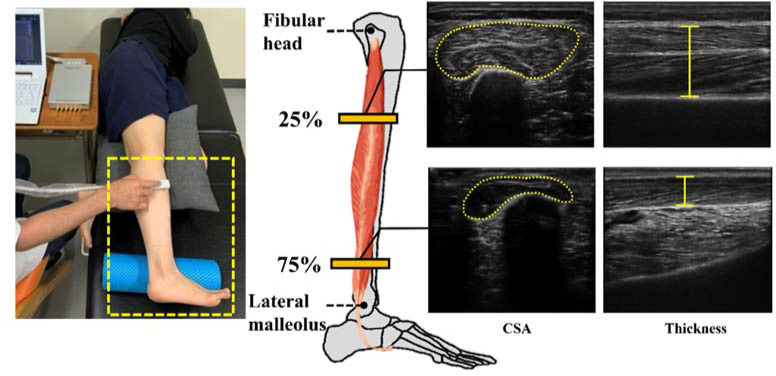
Peroneus muscles’ CSA, thickness, and echogenicity of the proximal 25% (indicates the morphology of the PL) and distal 75% (indicates the morphology of the PB) in a straight line connecting the fibular head and the lateral malleolus were measured using an ultrasound imaging system. PL: peroneus longus, PB: peroneus brevis, CSA: cross-sectional area

Figure 2 illustrates how the CSA, thickness, and echogenicity of the PL and PB were measured. We measured these parameters using a B-mode ultrasound imaging system (ArtUs EXT-1H, Telemed, Vilnius, Lithuania) equipped with a probe (frequency, 5–11 MHz; length, 60 mm; LF11-5H60-A3, Telemed). A single physical therapist with more than three years of experience with ultrasound imaging systems performed the measurements. We performed the measurements with participants lying in the lateral position on the bed. The measurement points were based on Arima et al. (2022), where the proximal 25% and distal 75% points of the straight line joining the fibular head and lateral malleolus corresponded to the PL and PB measurement points, respectively. The PL and PB CSAs were measured with the probe positioned vertically in the line joining the fibular head and lateral malleolus. The PL and PB thickness was measured at a position with the probe rotated 90° from the measurement position of the CSA. When obtaining the CSA and thickness images, the gel was applied to the probe sufficiently, and the probe was placed on the measurement point with minimal force. The measurement points were marked to ensure that the same position was used for each measurement. Echogenicity was calculated based on CSA images using ImageJ version 1.52 (National Institutes of Health, Bethesda, MD, USA) in a 8-bit grayscale with 256 steps, from 0 to 255. The average of three values from three images was used for each CSA, thickness, and echogenicity value. The lower the muscle echogenicity value, the lower the percentage of noncontractile tissues in the muscle, and the darker they appeared in ultrasound images (Ong et al., 2017).

### Ankle Strength Test Measurement

**Figure 3:**
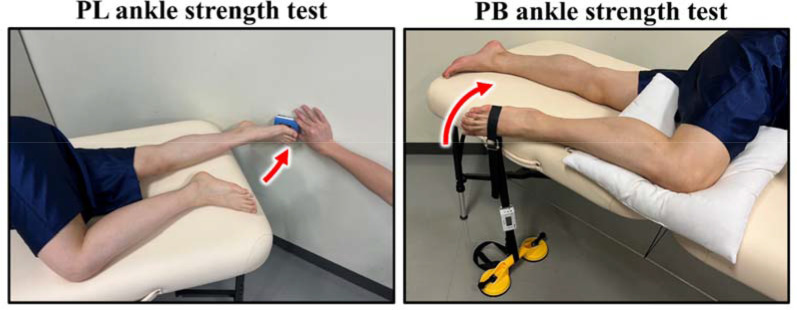
Two types of ankle strength tests were performed: a PL ankle strength test and a PB ankle strength test. PL: peroneus longus, PB: peroneus brevis

Figure 3 shows the PL and PB ankle strength test methods. We conducted two types of ankle strength tests: (1) the PL ankle strength test, in which isometric ankle strength was measured by pushing the handheld dynamometer (Mobie, Sakai Medical Co., Ltd., Tokyo, Japan) at the ball of the foot for 5 s with maximum effort from maximum ankle plantar flexion in the lateral position; and (2) the PB ankle strength test, which was performed by pulling the handheld dynamometer belt attached to the base of the fifth metatarsal with maximum effort for 5 s from maximum ankle plantar flexion in the lateral position (Ahn et al., 2020). In this study, ankle strength was defined as the value of the unidirectional output of the ankle obtained using a handheld dynamometer. The order of the ankle strength tests was randomly determined each time, and three measurements were taken for each type. We used the average value as the measured value. The values obtained from the handheld dynamometer were divided by the participant's body weight, and the ankle strength per body weight (N/kg) was used as the analytical value.

### Statistical Analysis

Statistical analyses were conducted using the Statistical Package for the Social Sciences (SPSS) version 28.0 for Windows (IBM Corp., Armonk, NY, USA). The normality and homoscedasticity of the measurement values for the CSA, thickness, and echogenicity at the proximal 25% and distal 75% locations and ankle strength of the PL and the PB were checked using the Shapiro-Wilk and Levene’s tests, respectively. If the assumption of sphericity was invalid, the Greenhouse-Geisser adjustment was applied. The intra-rater reliability of the CSA, thickness, and echogenicity at the proximal 25%, distal 75% locations and the ankle strength test values of the PL and the PB were determined using intra-class correlation coefficients (ICC) (1, 3). ICC values of <0.5, 0.5–0.75, 0.75–0.9, and ≥0.9 indicated low, moderate, good, and excellent reliability, respectively (Koo and Li, 2016). Additionally, the standard error of measurement and the minimum detectable change were calculated. To ensure no difference between the PL and PB intervention values at baseline, an unpaired *t*-test was used to evaluate all measurements. Two-way repeated-measures analysis of variance (ANOVA) was then performed to examine the differences in the effects of the 8-week PL and PB interventions on the CSA, thickness, and echogenicity at the proximal 25% and distal 75% locations, as well as ankle strength of the PL and the PB over time. Week (baseline and weeks 1–8, 10, and 12) was the within-group variable and the type of intervention (PL vs. PB intervention) was the between-group variable in this analysis. The value of the absolute change (Δ) from each week’s measurements compared to the baseline was used for all measurements. The main and interaction effects were evaluated for both within- and between-group variables. When a significant interaction was found, post hoc Bonferroni corrections were conducted to determine those time points which differed from the baseline. The level of significance was set at 5%, and the effect size was estimated using partial eta squared (ηp^2^); values of 0.01, 0.06, and 0.14 were indicative of small, medium, and large effect sizes, respectively (Richardson, 2011).

A post hoc power analysis was conducted using G*Power version 3.1.9.2 (Heinrich Heine University Düsseldorf, Düsseldorf, Germany) to assess the study’s statistical power and ensure that the sample size was adequate. The analysis aimed to determine whether the proximal 25% and distal 75% CSAs of the PL and the PB selectively hypertrophied over the 8-week intervention period, as indicated by the interaction between within-group (week) and between-group (type of intervention) variables. Thus, the analysis assessed the likelihood that any significant changes observed in the CSAs of the PL and the PB were due to interventions and not to chance. The smallest ηp^2^ value observed in the CSA interaction results was 0.526 for the distal 75% CSA, which was used to calculate the effect size (*f*) of 1.05. The other input parameters for the G*Power analysis were α err prob = 0.05, total sample size (n = 20), the number of groups (n = 2), the number of measurements (n = 11), Corr between rep measures = 0.5, and asphericity correction e = 1. The resultant power (1-β err prob) was 1.0, indicating sufficient statistical power to detect significant changes in the CSAs of the PL and the PB.

## Results

Table 2 presents the results of the ICC (1, 3) analysis for the CSA, thickness, and echogenicity at the proximal 25% and distal 75% locations and the ankle strength test values of the PL and the PB. All measurements displayed “almost perfect” reliability, with ICC (1, 3) values ranging from 0.81 to 1.00, according to the classification by Landis and Koch (1997) (kappa statistic = 0.81–1.00).

**Table 2:** ICC (1, 3) for the CSA and thickness and echogenicity at the proximal 25% and distal 75%, and PL and PB ankle strength

	ICC _1,3_	95% CI	SEM	MDC	*p*-value
**CSA (mm^2^)**					
Proximal 25%	0.97	0.95–0.99	11.31	31.35	<0.001
Distal 75%	0.95	0.90–0.98	9.74	26.99	<0.001
**Thickness (mm)**					
Proximal 25%	0.96	0.92–0.98	0.30	0.84	<0.001
Distal 75%	0.95	0.90–0.98	0.26	0.72	<0.001
**Echogenicity (a.u.)**					
Proximal 25%	0.95	0.90–0.98	1.27	3.53	<0.001
Distal 75%	0.92	0.84–0.96	2.10	5.81	<0.001
**Ankle strength (N/kg)**					
PL ankle strength	0.82	0.66–0.92	0.13	0.37	<0.001
PB ankle strength	0.85	0.72–0.93	0.21	0.57	<0.001

ICC: intraclass correlation coefficients, ICC (95% CI: confidence interval), SEM: standard error of measurement, MDC: minimal detectable change, PL: peroneus longus, PB: peroneus brevis, CSA: cross-sectional area

For all measurements, an unpaired *t*-test confirmed no differences in baseline values between the PL and PB interventions.

Table 3 summarizes the results of the two-way repeated-measures ANOVA. The significant effects of the week and the type of intervention were observed for the CSA at the proximal 25% and distal 75% locations and the ankle strength of the PL and the PB (*p* < 0.05). Additionally, interaction between the week and the type of intervention was significant for the CSA in the proximal 25% and distal 75% locations and ankle strength of the PL and the PB. However, no main effects of the week and the type of intervention and no interaction between the week and the type of intervention were found for thickness and echogenicity at the proximal 25% and distal 75% locations.

**Table 3:** Two-way repeated-measures ANOVA of peroneus muscles CSA, thickness, echogenicity at proximal 25% and distal 75%, and ankle strength

	Main effect (Week)			Interaction (Week*Type of Intervention)			
	F	*p*-value	ηp^2^		F	*p*-value	ηp^2^
**CSA (mm^2^**)							
Proximal 25%	107.062	<0.001	0.856		49.835	<0.001	0.735
Distal 75%	32.501	<0.001	0.644		19.942	<0.001	0.526
**Thickness (mm)**							
Proximal 25%	5.481	0.003	0.233		1.801	0.159	0.091
Distal 75%	3.892	0.028	0.178		2.425	0.100	0.119
**Echogenicity (a.u.)**							
Proximal 25%	2.779	0.070	0.755		0.395	0.918	0.305
Distal 75%	1.847	0.122	0.093		1.217	0.310	0.063
**Ankle strength (N/kg)**							
PL ankle strength	46.146	<0.001	0.719		19.111	<0.001	0.515
PB ankle strength	49.263	<0.001	0.732		23.009	<0.001	0.561

*p*-value < 0.05, PL: Peroneus longus, PB: Peroneus brevis, F: F-value, ηp^2^: Partial eta-squared. The within-group variable was week (baseline, weeks 1–8, 10, and 12) and the between-group variable was the type of intervention (PL intervention vs. PB intervention), CSA: cross-sectional area.

**Figure 4:**
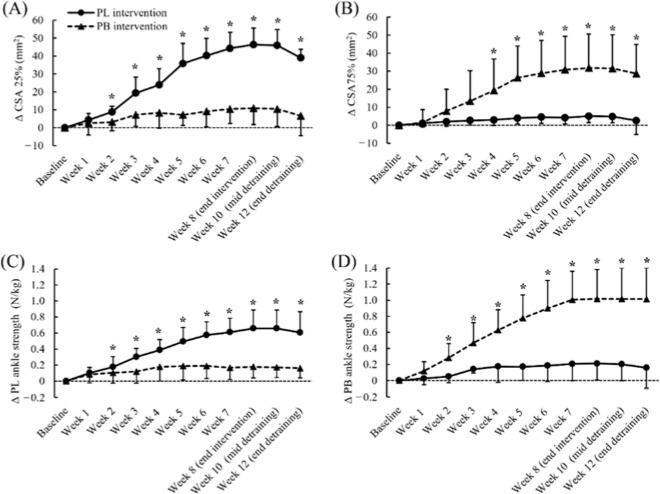
Results of absolute changes in (A) CSA 25%, (B) CSA 75%, (C) PL ankle strength, and (D) PB ankle strength during the 8-week longitudinal intervention and at weeks 10 and 12 of detraining. *p* < 0.05 (*): vs. baseline. All comparisons were made with baseline within the group. CSA: Cross-sectional area, PL: Peroneus longus, PB: Peroneus brevis, Δ: Absolute change from baseline

Figure 4 presents the post hoc Bonferroni correction results. The PL intervention group showed a significant increase in the proximal 25% CSA at weeks 2–8, 10, and 12 (*p* < 0.05), but not at week 1. The PB intervention group exhibited a significant increase in distal 75% CSA values at weeks 4–8, 10, and 12 (*p* < 0.05), but not at weeks 1–3. Furthermore, only the PL intervention group showed a significant increase in PL ankle strength values at weeks 2–8, 10, and 12 (*p* < 0.05), but not at week 1. Similarly, only the PB intervention group exhibited a significant increase in PB ankle strength values at weeks 2–8, 10, and 12 (*p* < 0.05).

## Discussion

We confirmed the viability of targeted training for the PL and the PB by examining the effects of 8-week selective training and the detraining period. Previous reports had examined the use of electromyostimulation to improve the function of the PL alone (Lima et al., 2022). However, no reports had focused on targeted training of the PL and the PB. This is the first study to examine whether long-term selective training of the PL and the PB is possible.

The main finding of the current study was the significant interaction between the week and the type of intervention for the CSA in proximal 25% and distal 75% locations and ankle strength values of the PL and the PB. This result implies that 8-week PL and PB interventions, including the post-intervention detraining period, caused distinct alterations in the morphology of these muscles and ankle strength. The post hoc comparison showed that, with each passing week, the proximal 25% CSA and PL strength values increased only following the PL intervention. The distal 75% CSA and PB strength values increased only with the PB intervention, demonstrating the robustness of the measurement protocol. Regarding the detraining effect, the proximal 25% CSA and PL strength test showed higher values at weeks 10 and 12, as compared to those at baseline, with PL intervention only. Likewise, the distal 75% CSA and PB strength test showed higher values with the PB intervention only. These findings confirm the persistence of the intervention effect for 8 weeks. It has been reported that the PL has the largest moment arm in ankle eversion, and the PB has the largest moment arm in ankle external rotation (McCullough et al., 2011). Accordingly, movements emphasizing ankle eversion, such as the PL intervention in our study, and movements emphasizing ankle external rotation, such as the PB intervention, were important for evaluating the selective intervention effects of PL and PB morphology and ankle strength. The training effect persisted to some extent even after the intervention period ended (De Souza et al., 2019). Therefore, we examined and confirmed the residual effect of the interventions during the detraining period. We observed that the CSA and ankle strength test values for the PL and the PB continually increased during the 8-week intervention period and were significantly higher than baseline values during the subsequent detraining period. These results suggest a high residual effect after training and that the training effect was maintained for four weeks after the cessation of the resistance training program.

In this study, we found a significant interaction between the training week and the type of intervention for the CSAs in both muscles; however, we found no interaction between these variables for muscle thickness, which did not confirm the long-term selective intervention effect. A previous study proposed that ultrasound muscle thickness assessments were only partially effective and did not completely reflect muscle hypertrophy (Franchi et al., 2018). Herein, the thickness characteristics that reflected local muscle hypertrophy would have prevented significant results for this measurement. Additionally, no significant difference was observed in muscle echogenicity between the PL and PB interventions, indicating that the intervention did not have a selective effect on muscle echogenicity. Several tissues, such as the connective and adipose tissues, likely affect muscle echogenicity (Strasser et al., 2013). It is possible that the intervention affected various tissues other than the muscles and did not alter muscle echogenicity. Therefore, when using ultrasound imaging systems to evaluate muscle hypertrophy caused by PL and PB training, the CSA, rather than thickness or echogenicity, is the recommended measurement, as the CSA provides a more comprehensive assessment of muscle hypertrophy.

There are several limitations in this study, which should be considered. First, the appropriate intervention period and load for PL and PB interventions are debatable. However, the results of this study confirmed the intended training effect, as a sustained muscle hypertrophy effect was observed during the detraining period after the 8-week intervention when muscle hypertrophy is typically observed. This study is significant because it demonstrated the possibility of selective training of the PL and the PB. Second, sex differences in training effects were not considered. A previous report noted that there might be a greater increase in muscle strength in women than in men, even with similar training loads (Roberts et al., 2020). Thus, additional studies with increased sample sizes and comparison of sex differences for selective training effects on the PL and PB are warranted to validate our findings. Finally, ankle strength was the output value from the handheld dynamometer in one direction of ankle motion. The ankle joint torque should be calculated using moment arms and muscle output (McCullough et al., 2011). Thus, to investigate PL and PB strength precisely, future research should include an examination of their respective moment arm and muscle output at that time, along with electromyographic measurements.

## Conclusions

In this study, we demonstrated that 8-week targeted training for the PL and the PB produced selective alterations in the CSA and ankle strength of these muscles over an extended period. The PL and the PB play distinct roles concerning the foot and the ankle. Therefore, it is important to evaluate muscle morphology and function of the PL and the PB individually instead of evaluating them collectively as peroneal muscles. Moreover, specific training in each of these muscles is necessary to improve the morphology and function of the PL and the PB following the injuries of the foot and the ankle, as well as to selectively train the PL and the PB in healthy individuals to enhance their muscle function and prevent injury over time. Our findings provide new insights into the application of targeted approaches to the foot and the ankle.
